# Corticosterone-mediated physiological stress modulates hepatic lipid metabolism, metabolite profiles, and systemic responses in chickens

**DOI:** 10.1038/s41598-019-52267-6

**Published:** 2019-12-17

**Authors:** Sarah J. M. Zaytsoff, Catherine L. J. Brown, Tony Montina, Gerlinde A. S. Metz, D. Wade Abbott, Richard R. E. Uwiera, G. Douglas Inglis

**Affiliations:** 1 Agriculture and Agri-Food Canada, 5403-1st Avenue S, Lethbridge, AB Canada; 2 Department of Agricultural, Food, and Nutritional Science, University of Alberta, 410 Agriculture/Forestry Centre, Edmonton, AB Canada; 3 Department of Biological Sciences, University of Lethbridge, 4401 University Drive, Lethbridge, AB Canada; 4 Department of Chemistry and Biochemistry, University of Lethbridge, 4401 University Drive, Lethbridge, AB Canada; 5 Department of Neuroscience, University of Lethbridge, 4401 University Drive, Lethbridge, AB Canada

**Keywords:** Fat metabolism, Metabolomics, Animal physiology

## Abstract

The impact of physiological stress on lipid metabolism, the metabolome, and systemic responses was examined in chickens. To incite a stress response, birds were continuously administered corticosterone (CORT) in their drinking water at three doses (0 mg/L, 10 mg/L, and 30 mg/L), and they were sampled 1, 5, and 12 days after commencement of CORT administration. Corticosterone administration to birds differentially regulated lipogenesis genes (*i.e. FAS*, *ACC*, *ME*, and *SREBF1*), and histopathological examination indicated lipid deposition in hepatocytes. In addition, CORT affected water-soluble metabolite profiles in the liver, as well as in kidney tissue and breast muscle; thirteen unique metabolites were distinguished in CORT-treated birds and this was consistent with the dysregulation of lipid metabolism due to physiological stress. Acute phase responses (APRs) were also altered by CORT, and in particular, expression of *SAA1* was decreased and expression of *CP* was increased. Furthermore, CORT administration caused lymphoid depletion in the bursa of Fabricius and elevated *IL6* and *TGFβ2* mRNA expression after 5 and 12 days of CORT administration. Collectively, incitement of physiological stress via administration of CORT in chickens modulated host metabolism and systemic responses, which indicated that energy potentials are diverted from muscle anabolism during periods of stress.

## Introduction

Poultry are subjected to numerous stressors throughout their lifetime. Temperature changes, social stress, and transportation are some consequences of the production cycle that can elicit a stress response in birds^1,2^. It has been demonstrated that physiological stress can impact production performance by altering various host functions. Meat quality, weight gain, and feed efficiency have been shown to be modulated by stress and are some examples of how stress can negatively impact production performance^3–5^.

Previous studies in poultry have demonstrated that physiological stress can result in increased expression of hepatic lipid synthesis genes and extrahepatic lipid deposition^4,6^. *De novo* lipogenesis primarily occurs in the liver and to a lesser extent in adipose tissue of poultry^7^. Fatty acid synthase (*FAS*) is a multifunctional enzyme that uses NADPH to catalyze malonyl-CoA into palmitate^8^. Malonyl-CoA is generated from the carboxylation of acetyl-CoA by acetyl-CoA carboxylase (*ACC*); additionally, this enzyme acts as the rate limiting step in fatty acid synthesis^9^. The hydrogen donor, NADPH, in *de novo* lipogenesis derives from the catalysis of malate by malic enzyme (*ME*) or from glucose catabolism in the pentose phosphate pathway^8^. Lipogenic gene expression is mediated by sterol regulatory element binding transcription factor (*SREBF1*) and liver nuclear X receptors^9^. Elevated levels of *SREBF1* corresponded with increased expression of cholesterol and fatty acid synthesis genes^9^. Synthesized lipids are then incorporated into very-low density lipoprotein (VLDL) for secretion from the liver where they enter circulation for deposition into extrahepatic and adipose tissue^10^.

The use of metabolomics in poultry research has increased in recent years and is currently being used to better understand a variety of host responses^11,12^. Examining the metabolome of tissues, blood, urine, and feces has created a novel method to assess the regulation of metabolic pathways. Furthermore, defined metabolite signatures that occur during disease can be used to develop novel biomarkers. Corticosterone (CORT) is a glucocorticoid released upon stimulation of hypothalamic-pituitary-adrenal axis in chickens and results in varying physiological changes needed to regain homeostasis^13^. Corticosterone treatment in broiler chickens has been shown to increase plasma levels of amino acids, suggesting modulations to protein metabolism^5^. Elucidating modulations to metabolism under physiological stress through the use of metabolomics may aid in the development of evidence-based and novel interventions. Such interventions could include feed additives or supplements that can be administered to birds under periods of stress and assist in restoring metabolic homeostasis.

Studies in cattle and swine have demonstrated an acute phase response (APR) can be elicited during physiological stress^14,15^. An APR involves the synthesis of acute phase proteins (APPs) in the liver that result in a systemic response to early inflammation^16,17^. The function of APPs in avian species is less defined than in mammals, although APPs in birds generally aid the host in restoring homeostasis and limiting microbial infection^18^. Ceruloplasmin (*CP*), transferrin (*TF*), and serum amyloid A1 (*SAA1*) are considered to be positive APPs in chickens with their expression increasing during an APR^18^. Zulkifli *et al*. demonstrated both *CP* and *TF* to be transiently elevated after CORT administration in chickens^19^. A stress-induced APR and changes to immune status are expected to be metabolically costly, and may be responsible for modifications to metabolic metrics observed during physiological stress in chickens. The majority of research elucidating the impact of glucocorticoids such as CORTs on immune function suggests that they act as anti-inflammatory agents and suppress adaptive immune responses^20^.

Understanding how metabolism and systemic responses are altered during physiological stress is essential to identifying mechanisms of reduced weight gain performance. We hypothesize that physiological stress will simultaneously modulate (i) hepatic lipid metabolism, (ii) the metabolome of breast muscle/kidney/liver tissues, and (iii) systemic physiological responses (i.e. acute phase and immune responses). To test these hypotheses, we administered CORT at two doses (10 mg/L and 30 mg/L) to birds through their drinking water as a method to simulate a stress response, and measured an array of host responses after 1, 5, and 12 days of CORT treatment. Histopathological changes and mRNA gene expression of lipid metabolism genes were measured in the liver as it is the primary site of lipid synthesis in birds^9^. Breast muscle, kidney, and liver tissues were subjected to metabolomic analysis to provide insights on how stress can modulate metabolite profiles and lead to biomarker discovery. The APR was examined through mRNA gene expression in the liver as this is the location where APP are synthesized^16^. Lastly, mRNA gene expression of immune genes and histopathologic changes were examined in the bursa of Fabricius as atrophy has been previously reported to occur as the result of corticosterone administration^21^.

## Results

### Corticosterone administration reduced weight gain efficiency

**Figure 1:**
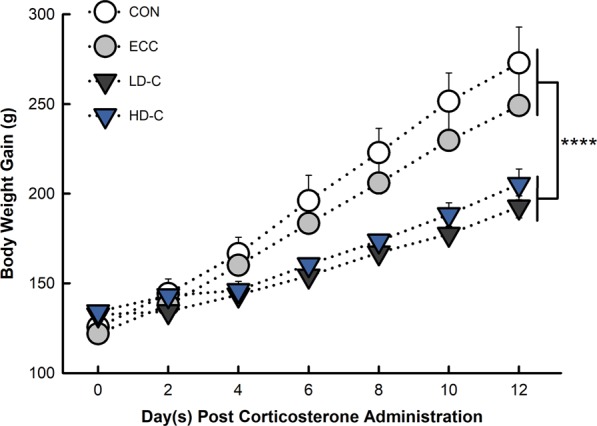
Effect of CORT treatment on body weight gain. Birds were administered standard drinking water (CON treatment), 0.2% ethanol in drinking water (ECC treatment), 10 mg/L CORT (LD-C treatment), or 30 mg/L CORT (HD-C treatment). Vertical lines associated with markers represent standard error of the means (n = 3); markers without vertical lines indicate that the marker is obscuring the standard error of the mean. ****p < 0.0001.

Weight gain was reduced (p < 0.0001) for the 10 mg/L low dose CORT (LD-C) treatment in comparison to both the non-CORT control (CON) and ethanol carrier control (ECC) treatments (Fig. 1). Weight gain was also reduced for the 30 mg/L high dose CORT (HD-C) treatment in comparison to the CON (p < 0.0001) and ECC (p = 0.006) treatments.

### Stress alters host physiology and hepatic histopathology

**Figure 2:**
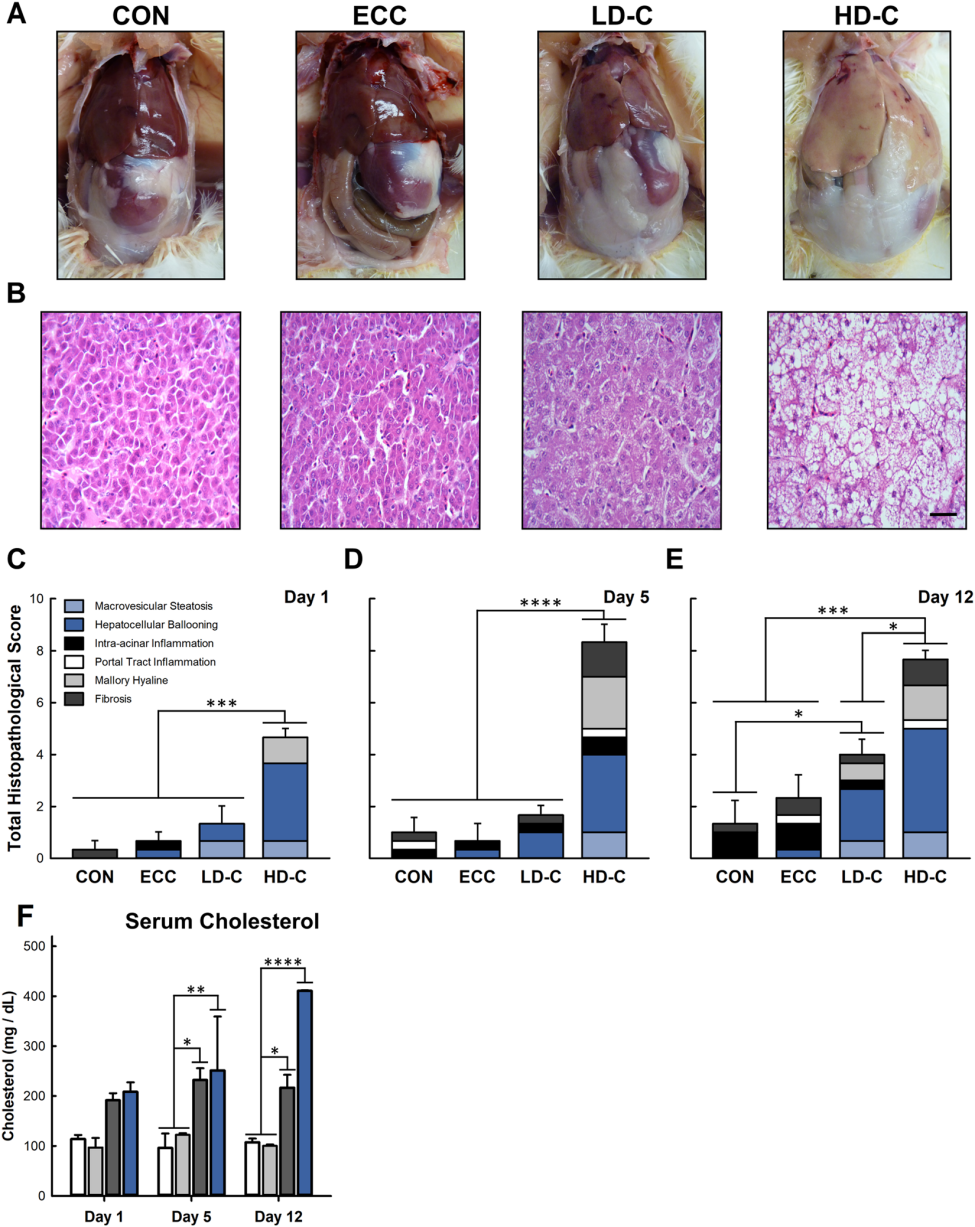
Effect of CORT treatment on host physiologic and histopathologic changes to the liver. Birds were administered standard drinking water (CON treatment), 0.2% ethanol drinking water (ECC treatment), 10 mg/L CORT (LD-C treatment), or 30 mg/L CORT (HD-C treatment); birds were euthanized and sampled at 1, 5, or 12 days post continual administration of CORT. **(A)** Gross morphology of liver and adipose tissue 12 days post-CORT administration. **(B)** Histopathological changes in liver tissue at 12 days post-CORT administration. Bar is 20 μm. **(C**–**E)** Total histopathological scores of liver. **(C)** 1 day post-CORT administration. **(D)** 5 days post-CORT administration. **(E)** 12 days post-CORT administration. **(F)** Serum cholesterol. Vertical lines associated with histogram bars represent standard error of the means (n = 3). *p < 0.05, **p < 0.01, ***p < 0.001, and ****p < 0.0001.

Gross morphological changes of the liver showed consistent enlargement and discolouration for the HD-C treatment 12 days post-CORT administration (Fig. 2A). Total histopathological scores of birds receiving HD-C were consistently increased after 1 day (p ≤ 0.001), 5 days (p ≤ 0.0001), and 12 days (p ≤ 0.0007) of CORT treatment in comparison to both the CON and ECC treatments (Fig. 2C–E). Only birds receiving the LD-C treatment for 12 days had elevated total histopathological scores in comparison to CON treatment birds (p = 0.031; Fig. 2E). Hepatocellular ballooning was a primary contributor to the elevated total histopathological scores observed for CORT treatment birds. The LD-C treated birds only demonstrated increased hepatocellular ballooning (p ≤ 0.025) after 12 days of CORT administration, where as the HD-C treated birds showed elevated hepatocellular ballooning after 1 (p ≤ 0.0009), 5 (p ≤ 0.0009), and 12 (p < 0.0001) days of CORT treatment in comparison to the CON and ECC treatments. The HD-C treatment also resulted in increased macrovesicular steatosis after 5 days (p < 0.023) of CORT treatment and elevated Mallory hyaline after 5 (p = 0.0027) and 12 (p = 0.023) days of CORT treatment. Serum cholesterol increased after 5 and 12 days of CORT treatment in birds for both the LD-C (p ≤ 0.038) and HD-C (p ≤ 0.01) treatments (Fig. 2F).

### Corticosterone modulates mRNA gene expression of hepatic lipid synthesis and secretion genes

**Figure 3:**
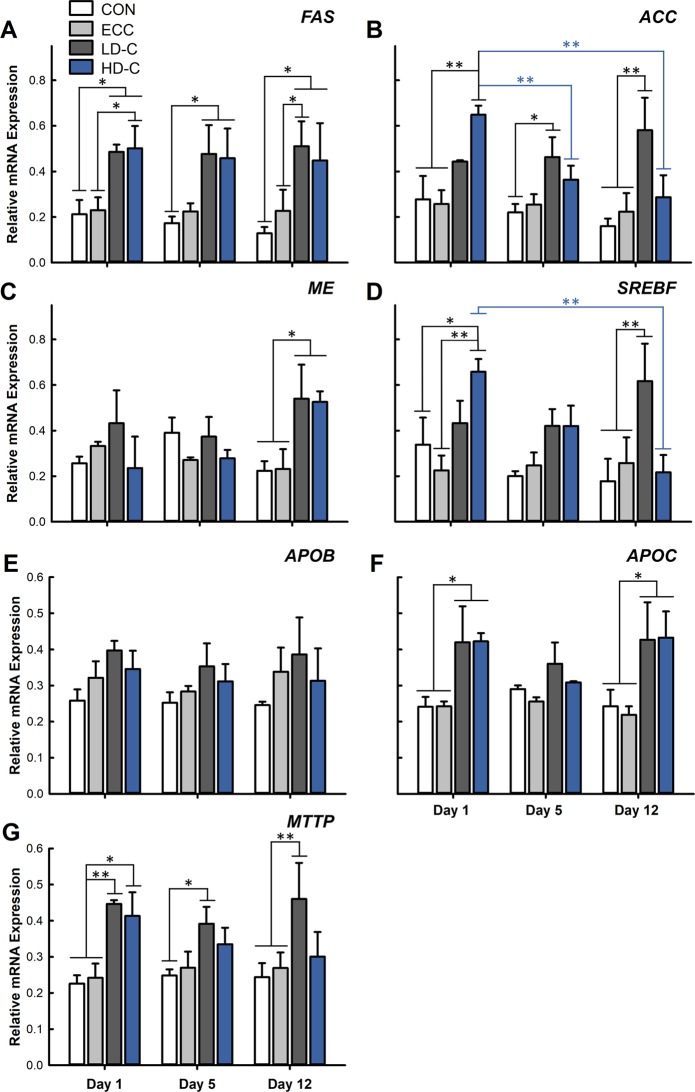
Corticosterone treatment modulates mRNA gene expression of lipid metabolism genes within the liver. Birds were administered standard drinking water (CON treatment), 0.2% ethanol drinking water (ECC treatment), 10 mg/L CORT (LD-C treatment), or 30 mg/L CORT (HD-C treatment); birds were euthanized and sampled at 1, 5, or 12 days post-CORT administration. **(A**–**G)** Relative expression of mRNA transcripts. **(A)** *FAS*. **(B)** *ACC*. **(C)** *ME*. **(D)** *SREBF1*. **(E)** *APOB*. **(F)** *APOC3*. **(G)** *MTTP*. Vertical lines associated with histogram bars represent standard error of the means (n = 3). *p < 0.05, **p < 0.01, ***p < 0.001, and ****p < 0.0001.

Birds receiving LD-C or HD-C demonstrated up-regulation of FAS 1 day (p = 0.045), 5 days (p = 0.038), and 12 days (p = 0.021) post-CORT administration relative to CON treatment birds (Fig. 3A). These results are consistent with the elevated *ACC* expression that was observed in birds administered CORT for 1 day at the high dose (p ≤ 0.0019). Only birds administered CORT at the low dose demonstrated elevated *ACC* expression after 12 days of CORT administration (p ≤ 0.0026). The HD-C treatment showed decreased *ACC* expression (p ≤ 0.013) after 5 and 12 days of CORT administration relative to expression levels after 1 day of CORT (Fig. 3B). The expression of *ME* was increased (p ≤ 0.023) after 12 days of CORT administration for both the LD-C and HD-C treatments (Fig. 3C). Increased expression of *SREBF1* was observed in birds receiving LD-C for 12 days (p ≤ 0.011), which supports upregulation of lipid synthesis (Fig. 3D). The expression of *SREBF1* was elevated (p ≤ 0.022) in HD-C-treated birds after 1 day of administration, but as observed with *ACC* expression, showed downregulation (p = 0.0025) after 12 days of CORT treatment (Fig. 3D). No changes in expression (p > 0.1) were observed for apolipoprotein B (*APOB*; Fig. 3E); however, increased (p ≤ 0.026) expression of apolipoprotein C3 (*APOC3*) was observed in birds receiving CORT after 1 and 12 days of administration (Fig. 3F). The expression of microsomal triglyceride transferase protein (*MTTP*) was increased (p ≤ 0.05) at all time points in birds administered CORT at a low dose in comparison to CON treatment. Birds administered CORT at a high dose only demonstrated elevated *MTTP* expression (p ≤ 0.026) at the 1 day post-administration time point (Fig. 3G).

### Corticosterone treatment alters metabolite profiles

**Figure 4:**
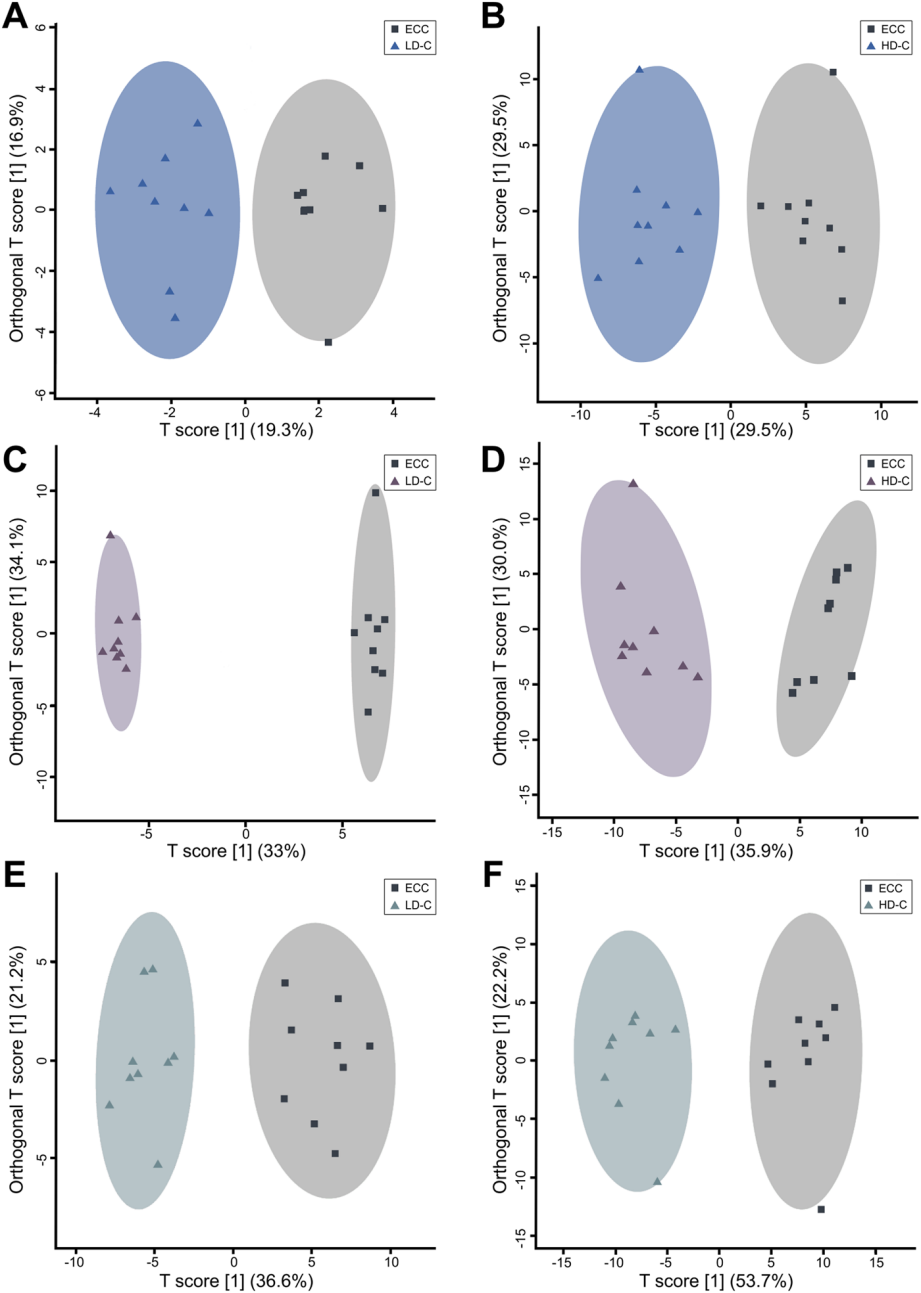
Metabolite profiles of kidney, liver, and breast muscle. OPLS-DA plots showing supervised separation for **(A**,**B)** kidney tissue, **(C**,**D)** liver tissue, and **(E**,**F)** breast muscle tissue, where (**A**,**C**,**E)** shows separation between birds administered 0.2% ethanol in drinking water (ECC treatment) or 10 mg/L CORT drinking water (LD-C treatment), and **(B**,**D**,**F)** shows separation between ECC treatment birds or 30 mg/L CORT of drinking water (HD-C treatment). Each square or triangle represents one bird; data was plotted using metabolites identified to be significant by MW and/or VIAVC. n = 9 birds per treatment.

**Figure 5:**
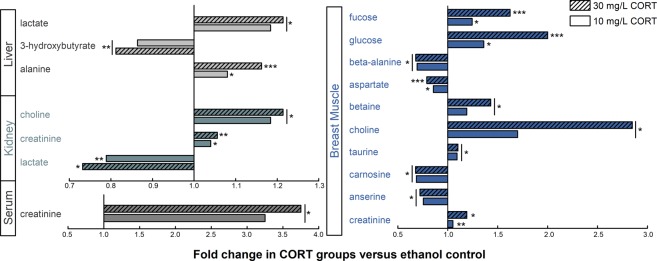
Fold-changes of discriminating metabolites due to CORT treatment. Metabolites were identified by ^1^H-NMR (liver, kidney, and breast muscle) and VetTest chemistry analyzer (serum). Individual metabolites and sample type are indicated; bars indicate fold changes of the normalized metabolome of birds receiving either 10 mg (LD-C treatment) or 30 mg (HD-C treatment) divided by the ethanol (ECC) treatment birds. n = 9 birds per treatment. *p < 0.05, **p < 0.01, ***p < 0.001.

Water-soluble metabolites were extracted from liver, breast muscle, and kidney tissues and analyzed by ^1^H-Nuclear Magnetic Resonance (NMR) spectroscopy to investigate metabolome changes associated with CORT administration. Analysis of both treatment groups included 439, 460, and 379 metabolite bins from the kidney, liver, and breast muscle, respectively. The LD-C and HD-C treatments were compared to ECC treatment birds to eliminate metabolite bias as a result of the ethanol carrier. Supervised OPLS-DA separation indicated that CORT administered at the low and high doses affected metabolite profiles in kidney (LD-C: p = 0.0005, Q^2^ = 0.709, R^2^ = 0.888; HD-C: p = 0.0005, Q^2^ = 0.888, R^2^ = 0.960), liver (LD-C: p = 0.004, Q^2^ = 0.846, R^2^ = 0.995; HD-C: p = 0.0005, Q^2^ = 0.804, R^2^ = 0.939), and breast muscle (LD-C: p = 0.001, Q^2^ = 0.665, R^2^ = 0.931; HD-C: p = 0.0005, Q^2^ = 0.904, R^2^ = 0.938) (Fig. 4A–F). Corticosterone administration significantly affected quantities of several metabolites in tissues (Fig. 5). In this regard, CORT treatment was associated with an increase (p ≤ 0.032) in lactate and alanine, and a decrease (p ≤ 0.0092) in 3-hydroxybutyrate in the liver. Kidney tissue showed that CORT administration resulted in an increase (p ≤ 0.031) in choline and creatinine, and a decrease (p ≤ 0.31) in lactate. In breast muscle, CORT administration was associated with an increase (p ≤ 0.018) in fucose, glucose, betaine, choline, taurine, and creatinine, and a decrease (p ≤ 0.035) in β-alanine, aspartate, carnosine, and anserine. Serum creatinine levels were elevated in CORT treatment birds (p ≤ 0.05).

### High dose corticosterone treatment is associated with histopathologic changes in the bursa of Fabricius

**Figure 6:**
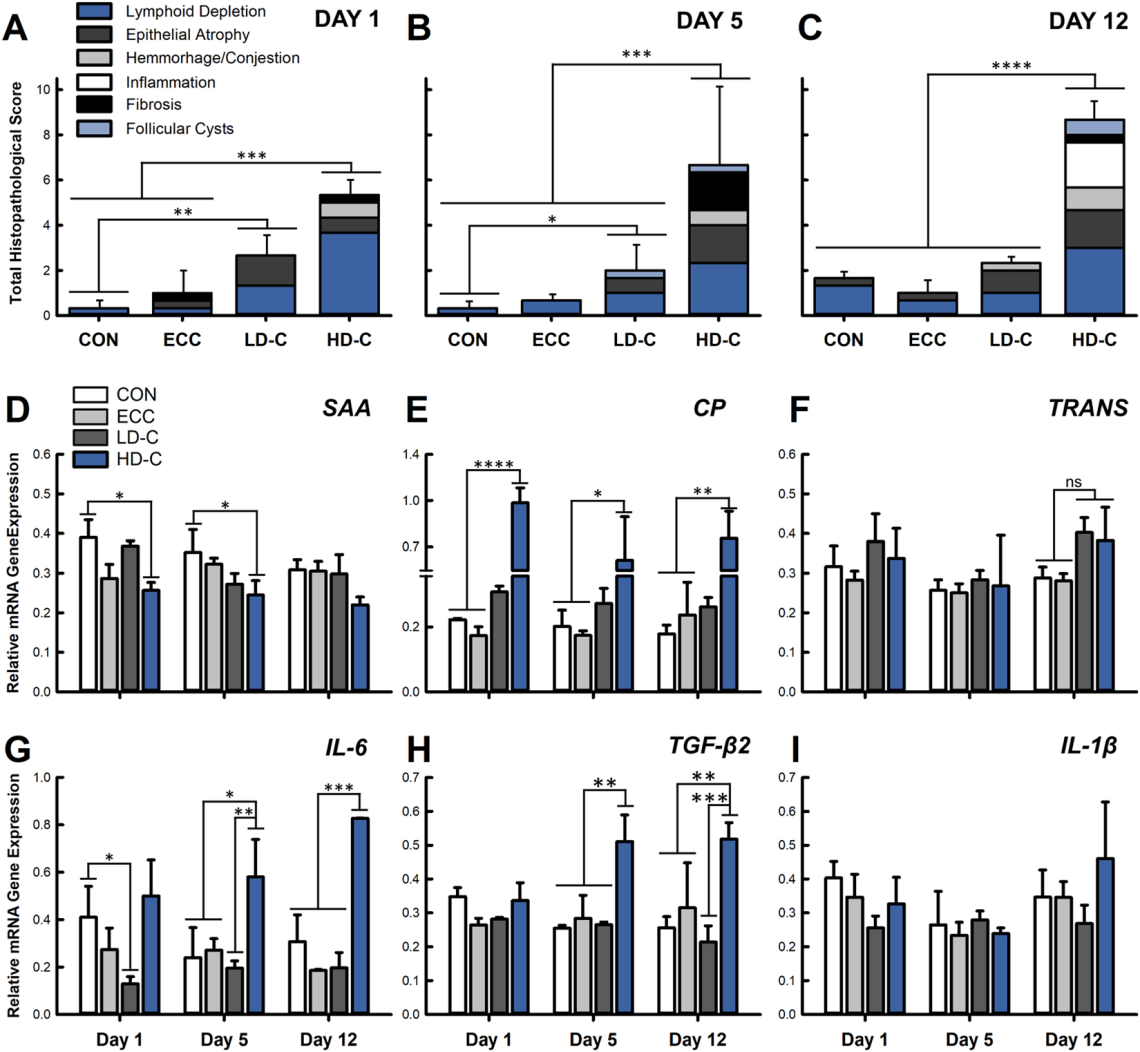
Effect of CORT treatment on systemic responses. Birds were administered standard drinking water (CON treatment), 0.2% ethanol drinking water (ECC treatment), 10 mg/L of CORT (LD-C treatment), or 30 mg/L of CORT (HD-C treatment); birds were euthanized and sampled at 1, 5, or 12 days post-CORT administration. **(A**–**C)** Total histopathologic change scores for bursa of Fabricius for **(A)** 1 day, **(B)** 5 days, or **(C)** 12 days post-CORT administration. **(D**–**I)** Relative mRNA gene expression in **(D**–**F)** liver and **(G**–**I)** bursa of Fabricius. **(D)** *SAA1*. **(E)** *CP*. **(F)** *TF*. **(G)** *IL6*. **(H)** *TGFβ2*. **(I)** *IL1β*. Vertical lines associated with histogram bars represent standard error of the means (n = 3). *p < 0.05, **p < 0.01, ***p < 0.001, and ****p < 0.0001.

Total histopathological scores of bursa of Fabricius tissues were higher in HD-C treatment birds at 1 (p ≤ 0.001), 5 (p ≤ 0.0001), and 12 (p ≤ 0.0001) days relative to ECC and CON treatment birds (Fig. 6A–C). Elevated scores in HD-C treatment birds were attributed to higher lymphoid depletion (day 1, p < 0.0001; day 5, p ≤ 0.035; day 12, p ≤ 0.041), epithelial atrophy (day 5, p ≤ 0.023; day 12, p ≤ 0.04), hemorrhage (day 12, p = 0.05), and after 12 days of CORT administration, due to elevated inflammation (p < 0.0027). No histopathologic changes (p ≥ 0.12) were observed in LD-C or ECC treatment birds relative to the CON treatment.

### Stress modulates mRNA gene expression of systemic responses

The expression of APPs in the liver and immune cytokines in the bursa of Fabricius were measured to identify systemic markers of a stress response. The administration of CORT modulated mRNA expression of APPs. The expression of *SAA1* mRNA decreased in HD-C treatment birds at the 1 day (p = 0.0096) and 5 days (p = 0.033) time periods relative to CON treatment birds (Fig. 6D). Ceruloplasmin mRNA expression was elevated (p ≤ 0.032) at all time points in HD-C treatment birds (Fig. 6E). Although a trend for increased expression of *TF* was observed after 12 days of HD-C treatment, this change was not defined as significant (p ≥ 0.1; Fig. 6F).

Corticosterone administration modulated immune cytokines in the bursa of Fabricius. A decrease (p = 0.05) in expression of Interleukin 6 (*IL6*) was observed after 1 day of LD-C administration, but remained unchanged (p ≥ 0.6) thereafter (Fig. 6G). Birds treated with HD-C showed increased expression of *IL6* after 5 (p ≤ 0.032) and 12 (p ≤ 0.0008) days of treatment in comparison to all other treatments (Fig. 6G). The expression of Transforming Growth Factor β2 (*TGFβ2*) was increased only in birds receiving HD-C after 5 (p ≤ 0.009) and 12 (p ≤ 0.018) days of treatment (Fig. 6H). The expression of Interleukin 1β (*IL1β*) showed no change (p = 0.59) due to CORT treatment (Fig. 6I).

## Discussion

Glucocorticoids, such as CORT, can have varying impacts on host physiologic, metabolic, and immunologic responses^3^. We examined the impact of administering CORT to chickens on hepatic lipid metabolism, metabolite compositions of liver, kidney and breast muscle, as well as a variety of systemic immune and stress responses. Data showed that CORT administration promoted lipid deposition in liver and resulted in distinct metabolite compositions in the liver, kidney, and breast muscle. Moreover, the expression of APPs in the liver and cytokines in bursa of Fabricius were modulated by CORT administration.

Gross and histopathological examinations showed that CORT administration resulted in discolouration of the liver, hepatocellular ballooning, and onset steatosis in birds 5 days after commencement of high dose corticosterone administration. Hepatic steatosis develops when the level of fatty acid synthesis exceeds the rate of secretion from the liver^10^. Furthermore, extrahepatic fattening is dependent on intravascular catabolism and release of lipids, which can then be incorporated into adipose tissue^7^. Our results are consistent with those of Jiang *et al*.^22^, who reported that corticosterone supplementation resulted in increased liver, cervical, and thigh fat deposition^22^.

Gene expression profiles of several hepatic lipogenesis genes were measured and the modulations observed corresponded to the histopathological findings of increased lipid deposition in the liver. Our results demonstrated that CORT administration resulted in elevated *FAS* expression after 1, 5, and 12 days of administration in both LD-C and HD-C treatment birds. Acetyl-CoA carboxylase was upregulated in LD-C or HD-C treatment birds after 1 day of CORT administration. Only birds receiving CORT at the low dose continued to show upregulation of *ACC* after 5 and 12 days of treatment. In contrast, *ACC* was down-regulated in HD-C treatment birds after 5 and 12 days of CORT administration. Acetyl-CoA carboxylase is negatively regulated by high cytosol levels of fatty acyl-CoA^23^, which may have contributed to the decreased expression of *ACC* that we observed in birds administered CORT at the high dose. The mRNA expression data corresponded to the histopathological data, which showed increased macrovesicular steatosis in HD-C treatment birds. The expression of *ME* was upregulated in birds receiving LD-C and HD-C, but only after 12 days of CORT administration. Unaltered *ME* expression after 1 and 5 days of CORT administration may indicate that the NADPH source needed for lipid synthesis was derived from other metabolic pathways (i.e. pentose phosphate pathway). The levels of *ME* can be altered by fasting and re-feeding states; given that the birds in this study were not starved at the time of sample collection, it is possible that *ME* expression may have been altered by the fed-state of the animals^24^. Birds administered HD-C showed elevated *SREBF1* expression after 1 day of CORT treatment, but like *ACC* expression, showed downregulation after 12 days of CORT administration. Conversely, birds administered LD-C showed elevated *SREBF1* expression only after 12 days of administration. Less is known about the *SREBF* family of proteins in poultry in comparison to mammals where several types of *SREBF* have been identified and have varying functions in the regulation of lipid and cholesterol synthesis^25^. Our results are in agreement with previous studies that showed that *FAS*, *ACC*, and *ME* were upregulated under dexamethasone mediated stress, and increased fat deposition under heat stress in chickens^6,26^. Furthermore, Cai *et al*.^6^ showed that dexamethasone administration resulted in higher levels of insulin which was associated with *de novo* lipogenesis^6,10^. Collectively, our findings indicate that lipid synthesis was upregulated in birds administered CORT, and that the expression of lipogenesis genes corresponded to gross morphological and histopathological changes.

The liver incorporates lipids into very-low density lipoproteins (VLDL), which are then exported into the bloodstream for delivery to adipose tissue and extrahepatic tissues. We measured two protein components of VLDL (i.e. *APOB* and *APOC3*)^27^. Additionally, we examined *MTTP*, which is responsible for assembly of VLDL and secretion from the liver^28^. No changes in gene expression of *APOB* were observed with CORT administration. Apolipoprotein B has been indicated as the major protein component of VLDL in chickens^10,27^, although the regulation and secretion of VLDL has not been the subject of much investigation in growing birds. *In vitro* studies have shown that high concentrations of insulin can enhance lipogenesis while concurrently inhibiting *APOB* synthesis. This can provide some explanation as to why *APOB* gene expression levels were unaltered by CORT administration in our study^10^. We observed that *APOC3* expression increased in birds administered CORT after 1 and 12 days. Little is known about the function of *APOC3* in avian species, and functional significance can only be currently derived from mammalian research. Interestingly, it has been demonstrated that *APOC3* can impair liver clearance of *APOB*, which corresponds to our findings on *APOB* expression^29^. Furthermore, *APOC3* has been shown to inhibit hepatic uptake of triglycerides^29^. In the case of our study, elevated *APOC3* can indirectly suggest that hepatocytes were saturated with lipids and further import would not be required. Lastly, *APOC3* has also been shown to promote VLDL assembly and secretion from hepatocytes under lipid rich conditions and this can promote expression of mRNA and function of *MTTP*^30^. This relates with our findings of *MTTP* gene expression, which demonstrated a similar expression pattern as *APOC3*. Both CORT treatments resulted in increased serum cholesterol at all sample time points. This indicates that circulating lipids were readily available for hydrolysis by lipoprotein lipase, a key enzyme necessary for the release of lipids for deposition into adipose tissue and extrahepatic tissues^31^. Only small amounts of lipoprotein lipase are found functionally active in poultry, and previous research has shown its gene expression is not upregulated under stress response^6,10^. Therefore, lipoprotein lipase was not measured in this study. The administration of CORT supported the up-regulation of genes involved in lipid synthesis in the liver, regardless of the duration of CORT administration (i.e. 1, 5, or 12 days). Increased lipid deposition suggests that energy distribution is affected during periods of stress in chickens, which may have been a contributing factor to reduced weight gain that was observed.

The use of metabolomics is becoming more widespread to investigate disease and identify biomarkers of physiological stress^32,33^. Our data demonstrated distinct metabolite compositions in tissue extracts of liver, kidney, and breast muscle that were associated with CORT administration (i.e. both LD-C and HD-C treatments). Our findings collectively demonstrate that physiological stress can alter several metabolic pathways and provides potential directions in the development of novel biomarkers of stress. In this regard, we observed increased creatinine in serum, kidney, and breast muscle of CORT-treated birds, which can indicate muscle breakdown and kidney dysfunction^34,35^. Heat stress in chickens has been demonstrated to increase creatine kinase activity; this enzyme is responsible for the breakdown of creatine and results in increased creatinine levels^2^. We observed decreased anserine and carnosine in muscle tissue of CORT-treated birds, which has been implicated as a marker of muscle dystrophy and altered redox homeostasis in chickens^33,36^. Furthermore, heat stress studies conducted in chickens and pigs concluded that decreased carnosine in muscle tissue lowered antioxidant capacity and this correlated with poor meat quality^37,38^. Likewise, high tissue concentrations of taurine are associated with elevated levels of oxidants and can aid in preventing tissue injury and inflammation^39^. A study in mice that examined the metabolome of muscle observed a correlation between increased choline, altered phospholipid metabolism, and membrane breakdown in muscle under high fat conditions^40^. High muscular choline levels observed in the current study may have derived from the hydrolysis of phosphatidylcholine present in VLDL. We observed an upregulation of *APOC3* and *MTTP* in CORT-treated animals, which suggests increased transport of VLDL to extremities of the body, such as muscle. Furthermore, the metabolites betaine and 3-hydroxybutyrate have been identified as discriminating metabolites of fat deposition, where betaine was increased and 3-hydroxybutyrate was decreased^11^. Similarly, lipid deposition mediated by CORT treatment in this study resulted in elevated betaine and decreased 3-hydroxybutyrate. We observed decreased β-alanine and aspartate levels, which may also be associated with CORT-induced lipid synthesis in the liver. Aspartate can be converted into β-alanine, which is subsequently metabolized into acetyl-CoA; however, this would be unnecessary in muscle tissue if lipid deposition is occurring and other energy sources are present^41^.

The sugars, fucose and glucose, were observed to be elevated in the breast muscle of CORT-treated birds. Fucose has previously been evaluated as a marker of liver disease where elevated urinary excretion of fucose was suggested to be a marker of impaired glycosylation in the liver^42^. Glucose may have been derived from two sources: (1) the breakdown of glycogen, which has been demonstrated under transport stress previously in chickens^4^; or (2) transport from the liver through the glucose-alanine cycle. The latter explanation is supported by our observation of increased alanine in the liver; alanine in the liver can be converted to glucose and transported back to muscle. However, glucose transporter 2 was not consistently upregulated in the liver with CORT treatments in our study, which would be expected if glucose transport to muscle was occurring (Supplementary Fig. S1). Conclusively, our results demonstrated that CORT-induced stress altered the metabolite profiles of liver, kidney, and breast muscle tissues and supported a stress-induced increase in hepatic lipid synthesis. Importantly, the conspicuous changes in metabolite compositions of tissues observed in birds administered CORT indicates dysregulation of metabolic pathways, and this likely contributed to the impaired weight gain observed.

We examined the expression of both acute phase proteins and immune cytokines as a measure of systemic response. Modulation of APP levels are often associated with inflammatory responses characterized by systemic and metabolic changes^17^. Bacterial agents, trauma, and other inflammatory mediators are often activators of APR. Acute phase proteins can have varying functions, but in general, act to restore homeostasis^17^. The expression of *SAA1* showed consistent decreases in HD-C treatment birds. Serum amyloid A is an apolipoprotein associated with high density lipoprotein and it is generally elevated under APR^43^. We observed a decreased in *SAA1* expression in CORT-treated birds, which may reflect alterations in hepatic lipid metabolism. The expression of *CP* was elevated in HD-C treatment birds at all sample times. Ceruloplasmin levels have been shown to be elevated in poultry proceeding infection with *Eimeria tenella* and *Escherichia coli*^44^. The function of *CP* is to catalyze iron into a ferrous state that can then be incorporated into iron binding proteins such as *TF*^18^. Higher levels of *TF* have been associated with inflammation where increased *IL6* levels and elevated heterophil numbers precede the increase of *TF*^45^. Increases in *TF* have been suggested to function by sequestering iron from bacterial utilization and thereby limiting infection. Our results however, did not demonstrate substantial increases in *TF* with CORT treatment.

The majority of studies conducted to date have primarily focused on how stress impairs immune response, specifically the generation of antibodies, or adaptive responses in chickens. Shini *et al*.^46,47^ examined cytokine and chemokine responses in immune cells, and showed that inflammatory responses can be activated during physiological stress. Our results demonstrated upregulation of *IL6* and *TGFβ2* in the bursa of Fabricius in HD-C treatment birds after 5 and 12 days of administration. Interleukin 6 can be produced in response to inflammation, and it is an inducer of APR, which corresponds to our findings of modulated APP expression^16^. Furthermore, Zulkifli *et al*.^19^ demonstrated that *IL6* serum levels were increased by CORT after 3 days of administration and persisted for 7 days. Another study demonstrated that *IL6*, along with pro-inflammatory cytokines *IL1β* and *IL18*, were upregulated 3 hr post-CORT administration in drinking water (20 mg/L) in peripheral lymphocytes and heterophils, while spleenocytes showed highest levels of these cytokines at 24 hr post-CORT exposure^46,47^. Although we did not observe an increase in *IL6* or *IL1β* expression after 1 day of CORT treatment, we measured mRNA expression in bursa of Fabricius tissue and did not examine expression within specific immune cell types. The bursa of Fabricius is a lymphoid organ unique to chickens and it is responsible for the development of B cells^17^. It is mainly comprised of B cells, some T cells, macrophages, and dendritic cells, which are organized into follicular structures^17^. We observed increased *IL6* expression within the bursa of Fabricius which may have been due to tissue degredation^19^. Our histopathological examination of the bursa of Fabricius showed that administration of CORT at the high dose induced epithelial atrophy and lymphoid depletion after 5 days of treatment with marked inflammation present after 12 days of CORT administration. Furthermore, the size of the bursa of Fabricius decreased with CORT treatment, and morphology was altered (Supplementary Fig. S2). Elevated *TGFβ2* mRNA that we observed in birds treated with CORT may have been due to tissue injury as *TGFβ* expression can be induced during tissue repair^48^. The histopathologic changes that we observed after 12 days of CORT administration support induction of inflammatory responses, as well as regulatory responses like *TGFβ2* due to physiological stress. Glucocorticoids can act to both activate and suppress immune responses^20^. We showed lymphoid depletion and atrophy of the bursa of Fabricius, which is consistent with the suppressive nature of glucocorticoids on immune function with respect to impaired adaptive immune responses. We concurrently observed an activation of *IL6* and *TGFB* expression which may have occurred within specific immune cells types as a response to tissue injury. Mammalian research has demonstrated that the suppressive or stimulatory nature of glucocorticoids on inflammatory responses can be dependant on both dose and duration^20^.

In summary, we demonstrated that CORT-mediated physiological stress resulted in higher expression of lipid synthesis and secretion genes, which led to increased lipid deposition in the liver. Metabolic perturbations observed in our study demonstrate the power of using metabolomic analysis to identify biomarkers of stress. Further research should collate metabolite changes in tissues to non-destructive markers (i.e. feces, feathers, blood) with the goal of achieving new diagnostic tools to better monitor on-farm stress. Our findings also showed that CORT treatment altered hepatic lipid metabolism, metabolite compositions in tissues, and APR after 1, 5, or 12 days of treatment. Likewise, modulations to cytokine expression in the bursa of Fabricius occurred after 5 and 12 days of CORT treatment were likely due to atrophy and other tissue damage. Collectively, the altered metabolic responses, APR induction, and immune modulations induced by CORT treatment likely contributed to the impaired weigh gain observed in birds. This has significant ramifications for poultry production, and additional research is required to investigate how poultry production stressors (i.e. thermal, social, etc.) can modulate immune function by examining cytokine profiles, immune cell trafficking, and paradigm immune responses. Crucially, obtaining a better understanding of how production stressors impact host immune and metabolic function is necessary to the identify and implement effective management regimes to enhance chicken health.

## Methods

### Experimental design

This study was designed as a factorial experiment with four levels of stress treatment and three levels of time arranged as a completely randomized design. The four stress treatments were: CON (untreated drinking water); ECC (0.2% ethanol drinking water); LD-C (10 mg corticosterone per 1 L of drinking water); and HD-C (30 mg corticosterone per 1 L of drinking water). Birds were euthanized and sampled from each stress treatment at 1, 5, and 12 days (n = 3 per treatment, n = 36 total). Each replicate included 12 chicks, and were conducted on three separate occasions to ensure independence.

### Ethics statement

The study was carried out in strict accordance with the recommendations specified in the Canadian Council on Animal Care Guidelines. The project was reviewed and approved by the Lethbridge Research and Development Centre (LeRDC) Animal Care Committee (Animal Use Protocol Review #1526) before commencement of the research.

### Birds

Thirty-six specific-pathogen-free white leghorn chickens were used in this study. Eggs were purchased from the Canadian Food Inspection Agency (Ottawa, ON). Eggs were incubated in a Brinsea Octagon 40 Advanced Digital Egg Incubator according to the manufacturer’s guidelines for incubating chicken eggs (Brinsea Products Inc., Titusville, FL). Eggs were maintained at 37.5 °C and 45% humidity with hourly turning of the eggs for the first 18 days of incubation. Thereafter, eggs were set flat for hatching and humidity was increased to 60%. All hatched chicks were acclimatized in a group within one large animal pen (1.1 m^2^) for 10 days, and had access to a brooder (Brinsea Products Inc.). Birds had *ad libitum* access to a non-medicated starter diet (Hi-Pro Feeds, Lethbridge, AB) and water at all times. Birds were maintained on a 12 hr light: 12 hr dark cycle. At 11 days-of-age, birds were randomly assigned to the four stress treatments and housed in groups of four within an individually ventilated cage (IVC) (Techniplast, Montreal, QC). Each animal cage contained a companion bird to ensure no birds were socially isolated. Birds were weighed daily throughout the study.

### Corticosterone administration

Corticosterone treatment occurred as previously described^49^. Birds began receiving CORT when chicks reached 14-days-of-age and continued until the end of the experiment. Corticosterone (Sigma Aldrich Inc., Oakville, ON) was dissolved in 2.0 mL of anhydrous ethanol and added to 1 L of drinking water. Water containing corticosterone was prepared fresh each day, and added to animal cages twice daily.

### Animal euthanasia and sample collection

One bird per stress treatment was randomly sampled on day 1, 5, or 12 after initiation of corticosterone treatment. Birds were anaesthetized with isoflurane (5% isoflurane; 1L O_2_/min), and blood was collected by intracardiac puncture. Birds were then euthanized by cervical dislocation under general anaesthesia. The abdomen was opened with a ventral midline incision, and the viscera was examined grossly for changes in morphology. The liver, kidney, breast muscle, jejunum, and bursa of Fabricius were aseptically removed, and the bursa of Fabricius was weighed. Samples for RNA analysis were immediately placed within RNAlater^®^ (Qiagen Inc., Toronto, ON). Tissues for histopathology were placed in 10% neutral buffered formalin. All remaining samples were stored at −80 °C until processing.

### Serum analysis

Concentrations of creatinine and cholesterol were measured using VetTest Chemistry Analyzer (Idexx Laboratories, Westbrook, ME). Dry slide technology panels specific to creatinine and cholesterol were used and the manufacturer’s guidelines were followed for metabolite quantification.

### Histopathology

Liver and bursa tissue samples were fixed in 10% neutral buffered formalin for minimum 24 hr. Samples were then dehydrated and embedded in paraffin blocks. Tissues were then sectioned (5 μm) using a microtome (Thermo Scientific™, Cheshire, UK), de-paraffinized with xylene, and stained with hematoxylin and eosin. Tissue sections were scored by a veterinary pathologist (R.R.E.U) blinded to treatments using modified scoring criteria previously described^50,51^. Three tissue sections for each animal and tissue type were examined to ensure uniformity of observations. Representative tissue sections were scored from a single field of observation within each histological section. Liver tissues were graded 0 to 4 for hepatocellular ballooning and fibrosis; 0 to 3 for macrovesicular steatosis, intra-acinar inflammation, portal tract inflammation, and Mallory’s hyaline. Bursa tissues were graded 0 to 4 for lymphoid depletion, epithelial atrophy, hemorrhage/congestion, inflammation, fibrosis, and follicular cysts. Total pathologic changes were determined by calculating the sum of scores from all tissue assessment criteria for each bird.

### Quantification of mRNA gene expression

Procedures for RNA extraction, reverse transcription, and quantitative PCR were followed as previously described^52^. Total RNA was extracted from liver, bursa of Fabricius, and distal jejunum tissue using a RNeasy mini kit (Qiagen Inc.) according to the manufacturer’s instructions. An additional DNase treatment (Qiagen Inc.) to the extraction column was included to remove residual genomic DNA. A Bioanalyzer RNA 6000 Nano kit (Agilent, Mississauga, ON) was used to measure RNA integrity and quantity, and 1.0 μg of RNA was reverse transcribed to cDNA using QuantiTect reverse transcription kit (Qiagen Inc.). Reactions were run on a 384-well plate, where each reaction contained 5.0 µl QuantiTect SYBR Green Master Mix (Qiagen Inc.), 0.5 µl of each primer (10 µM), 3.0 µl of RNase-free water, and 1.0 µl of cDNA. Quantitative PCR was performed using an ABI7900HT thermocycler (Applied Biosystems, Carlsbad, CA) with the following PCR conditions: 95 °C for 15 min; 40 cycles of 95 °C for 15 sec, 58–60 °C for 30 sec, and 72 °C for 30 sec; and melt curve analysis from 55–95 °C. Primer sequences specific to gene targets (Table S1) were generated using NCBI Primer-BLAST; primers were designed to create an amplicon between 75 and 200 base pairs. Efficiencies for all primers were between 95–110% and a single peak was present in melt curve analysis. Reactions were run in triplicate and average cT values were used to calculate gene expression relative to two reference genes (*Ba* and *Tbp)* using geNorm and qBase + software (Biogazelle, Gent, Belgium) quantification model^53^.

### Tissue metabolomics

Tissue samples were processed as outlined in Wu *et al*.^54^ with the following modifications. Liver, breast muscle, and kidney tissue was suspended in 4.0 mL/g methanol and 1.6 mL/g deionized water. Tissues were then homogenized with a 6-mm-diameter steel bead for 5 min using a Qiagen TissueLyser LT at 50 Hz followed by 1 min of vortexing. This step was repeated two times to ensure complete tissue homogenization. To each sample, 2.0 mL/g chloroform was added and vortexed thoroughly. Next, 2.0 mL/g chloroform and 4.0 mL/g deionized water was added to each sample and vortexed until thoroughly mixed. Samples were then incubated at 4 °C for 15 min followed by centrifuging at 1000 × g for 15 min at 4 °C, and 700 μl of the supernatant was placed into a microfuge tube and left for 3–4 days to air dry. Samples were rehydrated in 480 μl metabolomics buffer (0.125 M KH_2_PO_4_, 0.5 M K_2_HPO_4_, 0.00375 M NaN_3_, and 0.375 M KF; pH 7.4). A 120 μl aliquot of deuterium oxide containing 0.05% v/v trimethylsilylpropanoic acid (TMSP) was added to each sample (final total volume of 600 μl); TMPS was used as a chemical shift reference for ^1^H-NMR spectroscopy. A 550 μl aliquot was then loaded into a 5 mm NMR tube and run on a 700 MHz Bruker Avance III HD spectrometer (Bruker, Milton, ON) for spectral collection. Data acquisition and processing were followed as previously described^55^. MATLAB (MathWorks, Natick, MA) was used for spectral peak alignment and binning using Recursive Segment Wise Peak Alignment^56^ and Dynamic Adaptive Binning^57^, respectively. The dataset was then normalized to the total metabolome, excluding the region containing the water peak, and pareto scaled. Metaboanalyst^58^ was utilized for both the Orthogonal Partial Least Squares Discriminant Analysis (OPLS-DA) model and the calculation of fold changes in specific metabolites. Metabolites were identified using Chenomx 8.2 NMR Suite (Chenomx Inc., AB, Canada).

### Statistical analysis

Statistical analyses for gene expression, histopathology tissue scores, and weight gain were performed using Statistical Analysis Software (SAS Institute Inc. Cary, NC). Main effects within the experimental design were stress (n = 4) and time (n = 3) arranged as a completely randomized design. Gene expression data was assessed for normality and analyzed by a mixed linear model using the MIXED procedure of SAS. Body weight measurements were treated as a repeated measure; the appropriate covariance structure was utilized according to the lowest Akaike’s Information criterion. In the event of a main treatment effect (p ≤ 0.050), the least squares means test was used to compare treatments within factors for body weight and gene expression analysis. Histopathologic data (i.e. categorical data) was analyzed using a non-parametric Fisher’s exact test within SAS; pairwise comparisons were performed among stress treatments at the 1, 5, and 12 day time points. Metabolomics analysis was performed using MATLAB (MathWorks). Spectral bins were subjected to both univariate and multivariate analysis to determine which metabolites were significantly altered. The univariate measures were calculated using a decision tree algorithm as described by Goodpaster *et al*.^59^. The multivariate tests utilized the Variable Importance Analysis based on random Variable Combination (VIAVC) algorithm, which combines both Partial Least Squares Discriminant Analysis (PLS-DA) and the area under the Receiver Operating Characteristics (ROC) curve to synergistically determine the best subset of metabolites for group classifications^60^. All p-values obtained for metabolomic analyses were Bonferroni-Holm corrected for multiple comparisons; orthogonal Partial Least Square Discriminate Analysis (OPLS-DA) was carried out using the bins identified as significant by univariate and/or multivariate testing in order to observe group separation. Data is represented by mean ± standard error of the mean (SEM). Significance is indicated as *p < 0.05, **p < 0.01, ***p < 0.001, and ****p < 0.0001.

## Supplementary information

Dataset 1
