# Supplementary material for: Corticosterone-mediated physiological stress modulates hepatic lipid metabolism, metabolite profiles, and systemic responses in chickens
Source: Sci Rep. 2019 Dec 17;9:19225. doi: 10.1038/s41598-019-52267-6 (PMC6917734; doi:10.1038/s41598-019-52267-6)
Supplement: Supplementary file 1 — Dataset 1 [file 41598_2019_52267_MOESM1_ESM.pdf]

Corticosterone-mediated physiological stress modulates hepatic lipid metabolism, metabolite profiles, and systemic responses in chickens

Sarah J.M. Zaytsoff[1,2], Catherine L. J. Brown[1,3], Tony Montina[4], Gerlinde A.S. Metz[5], D. Wade Abbott[1], Richard R.E. Uwiera[2] & G. Douglas Inglis[1]

Figure S1

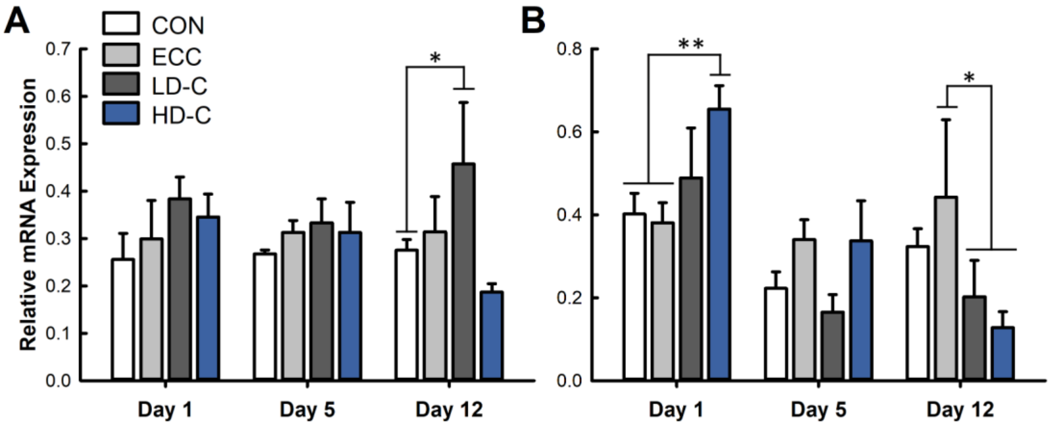

Figure S1. Effect of CORT treatment on *GLUT2* mRNA expression. Birds were administered standard drinking water (CON), 0.2% ethanol drinking water (ECC), 10 mg per litre of CORT (LD-C), or 30 mg per litre of CORT (HD-C); birds were euthanized and sampled at 1, 5, or 12 days post continual treatment. **(A-B)** relative mRNA gene expression of *GLUT2* in **(A)** liver and **(B)** jejunum**.** Bars represent mean (n=3) and vertical lines associated with bars represent standard error of the mean. * $p<0.05$ and ** $p<0.01$.

Figure S2

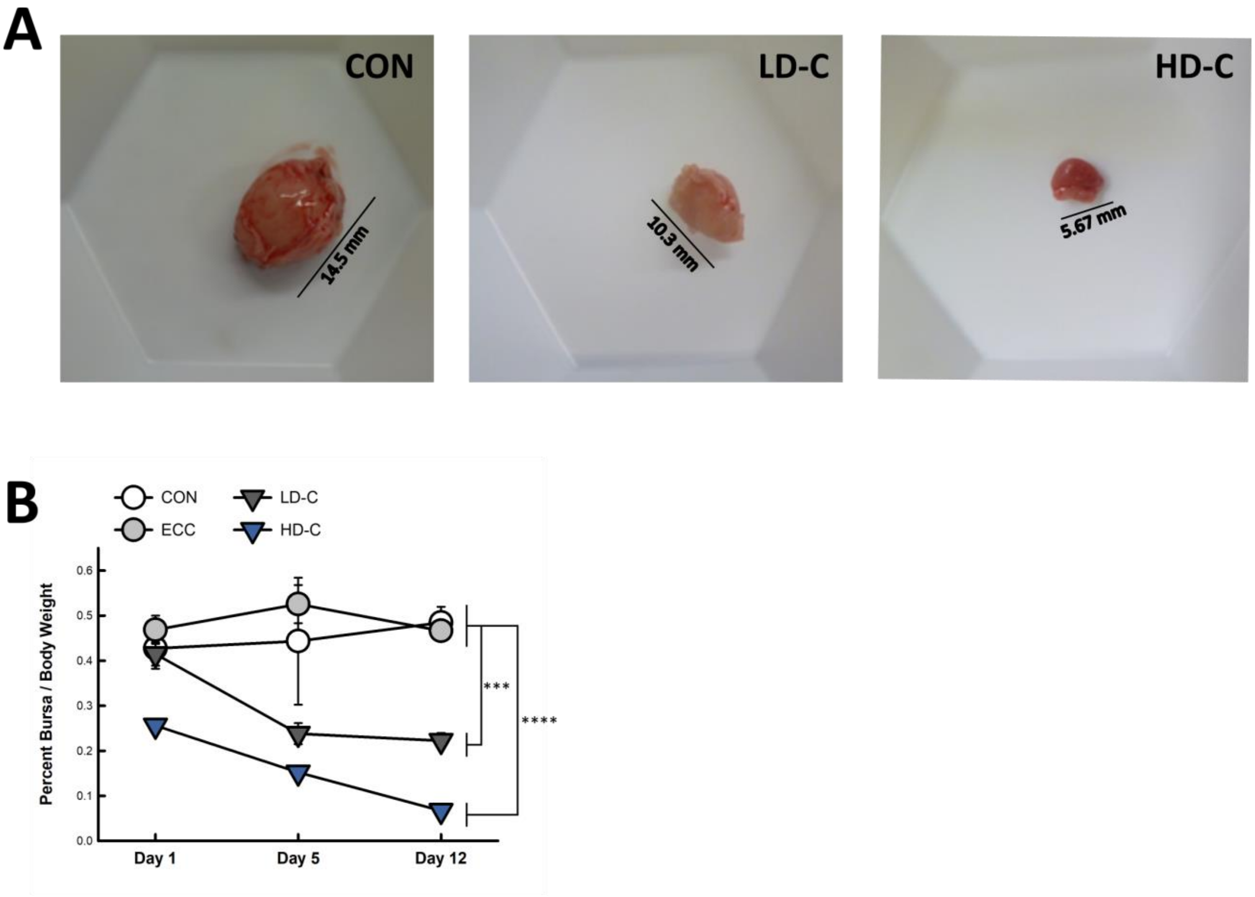

Figure S2. CORT treatment reduces size of bursa of Fabricius. Birds were administered standard drinking water (CON), 0.2% ethanol drinking water (ECC), 10 mg per litre of CORT (LD-C), or 30 mg per litre of CORT (HD-C); birds were euthanized and sampled at 1, 5, or 12 days post continual treatment. **(A)** gross morphology of bursa of Fabricius after 12 days of CORT administration. **(B)** relative size of bursa of Fabricius to body weight. Markers represent mean (n=3) and vertical lines associated with markers represent standard error of the mean. Markers without vertical lines indicates marker is obscuring the standard error of mean. *** $p<0.001$ and **** $p<0.0001$.

Table S1. List of primers used for mRNA gene expression

Name	Abbreviation	Sequence 5' to 3'		Product Size	Reference Sequence	Source
Fatty Acid Synthase	*FAS*	Forward	CAACAGCCAGCTTGGAATGG	161	NM_205155.2	This Study
		Reverse	CTGTGGTTCTCAGGGGCTTT			
Acetyl-CoA Carboxylase	*ACC*	Forward	CGTGAGGAGCCCATTCACAT	171	NM_205505.1	This Study
		Reverse	TGGAAATTCCCTCTTCTGTGC			
Malic Enzyme	*ME*	Forward	AATACACAGAGGGACGTGGC	121	NM_204303.1	This Study
		Reverse	GCAACTCCAGGGAACACGTA			
Sterol Regulatory Element Binding Factor 1	*SREBF1*	Forward	CAGAAGAGCAAGTCCCTCAAG	129	NM_204126.2	This Study
		Reverse	GGAGCCTACATCCGAGGG			
Apolipoprotein B	*APOB*	Forward	AGCCAAACTAAGTGGACTGC	100	NM_001044633.1	This Study
		Reverse	GATCCGGCCTTCACTTTTCA			
Apolipoprotein C-III	*APOC3*	Forward	CAAGCCAGGAAATGGCTGTC	184	NM_001302127.1	This Study
		Reverse	GACAAAAGGGAAACGGTGCT			
Microsomal Triglyceride Transferase Protein	*MTTP*	Forward	AAACTGGAACTGCAATCGGT	101	NM_001109784.2	This Study
		Reverse	GGCATGGAAACGTAACTGGA			
Glucose Transporter 2	*GLUT2*	Forward	GGAGGCCAAAAAGAGTTTGAAG	105	NM_207178.1	This Study
		Reverse	ACTCTCTTTTCACTCGCAGC			
Serum Amyloid A1	*SAA1*	Forward	TGGGTGAAGTGGTGGACAAA	180	XM_003641328.3	This Study
		Reverse	CTGGACTATCTGAGCGGACT			
Ceruloplasmin	*CP*	Forward	GGGCTTAGGGTCAGAAGTCG	147	XM_015291853.1	This Study
		Reverse	TCAAAAATTCCTTCAGAATCAGGCT			
Transferrin	*TF*	Forward	TACTTCAGCGAGGGTTGTGC	148	NM_205304.1	This Study
		Reverse	CCAGACACCGTAAAGCTCCG			
Interleukin 1β	*IL1β*	Forward	TGCCTGCAGAAGAAGCCTCG	137	NM_204524.1	This Study
		Reverse	CTCCGCAGCAGTTTGGTCAT			
Interleukin 6	*IL6*	Forward	AGGACGAGATGTGCAAGAAG	176	NM_204628.1	This Study
		Reverse	CATTTCTCCTCGTCGAAGCC			
Transforming Growth Factor β2	*TGFβ2*	Forward	CCATCTACAACAGCACCAGGG	157	NM_001031045.3	This Study
		Reverse	TAGCTTGGTGGGATGGCATTTTC			
β-Actin	*BA*	Forward	CTCTGACTGACCGCGTTACT	172	NM_205518.1	This Study
		Reverse	TACCAACCATCACACCCTGAT			
Tata-Box Binding Protein	*TBP*	Forward	GTTCCCTGTGTCGCTTGC	147		PMID: 27685470
		Reverse	TAGCCCGATGATGCCGTAT			
